# Comparative Phytochemical and Antiviral Characteristics of *Dipsacus asperoides* and *Phlomis umbrosa* Against Influenza A Virus: LC-QTOF-MS Profiling and Exploratory Network Pharmacology

**DOI:** 10.3390/plants15172649

**Published:** 2026-08-29

**Authors:** Ji Sun Park, Seung Mok Ryu, Jun Lee, Sungyu Yang, Hyo-Seon Kim, Young-Hye Seo, Hyun Ji Lee, Inkyu Park, Sohee Jeong, Jinseok Jung, Su-Jin Park, Joong Sun Kim

**Affiliations:** 1 Functional Biomaterial Research Center, Korea Research Institute of Bioscience and Biotechnology, Jeongeup 56212, Republic of Korea; jsp5928@kribb.re.kr (J.S.P.); honse5@kribb.re.kr (J.J.); 2 College of Veterinary Medicine and BK21 FOUR Program, Chonnam National University, Gwangju 61186, Republic of Korea; 3 Herbal Medicine Resources Research Center, Korea Institute of Oriental Medicine, Naju 58245, Republic of Korea; smryu@kiom.re.kr (S.M.R.); junlee@kiom.re.kr (J.L.); sgyang81@gmail.com (S.Y.); hs0320@kiom.re.kr (H.-S.K.); wnsl1118@kiom.re.kr (Y.-H.S.); hyunji@kiom.re.kr (H.J.L.); 4 Department of Biology, Changwon National University, Changwon 51140, Republic of Korea; pik6885@changwon.ac.kr; 5 Animal Medical Institute, Chonnam National University, Gwangju 61186, Republic of Korea

**Keywords:** *Dipsacus asperoides*, *Phlomis umbrosa*, Sokdan, Influenza A virus, comparative phytochemistry, LC-QTOF-MS, antiviral response, exploratory network pharmacology

## Abstract

**Background:** Influenza A virus (IAV) remains an important respiratory pathogen, and medicinal plants continue to provide chemically diverse sources for antiviral research. *Dipsacus asperoides* and *Phlomis umbrosa* have both been used under the traditional name ‘Sokdan’ despite their distinct botanical origins and phytochemical compositions. This study comparatively characterized their chemical profiles and in vitro responses to IAV and used network pharmacology as an exploratory approach to identify candidate pathways for subsequent mechanistic studies. **Methods:** Ethanolic extracts were evaluated for cytotoxicity in MDCK cells, cytopathic effect (CPE) reduction against IAV A/PR/8/34 (H1N1), and neuraminidase (NA) inhibition. LC-QTOF-MS was used for qualitative chemical profiling, and species-specific compound sets were subjected to target prediction, protein–protein interaction analysis, and pathway enrichment. **Results:** *D. asperoides* was non-cytotoxic up to 100 µg/mL and *P. umbrosa* up to 300 µg/mL under the tested conditions. Both extracts produced modest CPE reduction, reaching maximum inhibition of 21.8 ± 1.4% at 100 µg/mL for *D. asperoides* and 17.1 ± 6.1% at 200 µg/mL for *P. umbrosa*, whereas zanamivir (5 µM) produced 76.2 ± 0.8% inhibition. NA inhibition was limited for both extracts. LC-QTOF-MS identified twelve constituents with distinct species-associated detection patterns, including phenylpropanoids, iridoid glycosides, and triterpenoid saponins. Exploratory network analysis identified partially overlapping predicted hubs, including MAPK1/3, RELA, IL-6, TNF, and CASP3, together with additional species-specific nodes. **Conclusions:** Despite clearly different phytochemical profiles, the two Sokdan species showed a shared pattern of modest in vitro antiviral response. The network pharmacology results should be interpreted as hypothesis-generating rather than mechanistic confirmation and suggest candidate host-response pathways that can guide targeted follow-up experiments. These findings support phytochemical differentiation of the two species while providing a comparative framework for future investigation of their antiviral properties.

## 1. Introduction

Influenza A virus (IAV) is a negative-sense, single-stranded RNA virus belonging to the family Orthomyxoviridae and remains one of the foremost causes of acute respiratory morbidity and mortality worldwide [1,2]. The annual epidemics and occasional pandemics caused by IAV arise primarily from its high rates of antigenic drift caused by the error-prone viral RNA polymerase and antigenic shift, through which novel reassortant strains emerge [3]. Vaccination remains the most effective prophylactic measure; however, rapid antigenic changes in the major surface glycoproteins, hemagglutinin (HA) and neuraminidase (NA), necessitate annual vaccine reformulation and limit long term protective efficacy [4].

Currently approved antiviral strategies primarily target the viral NA (zanamivir, oseltamivir) or the viral RNA polymerase complex (baloxavir marboxil). Although these agents have significantly reduced influenza-related morbidity, the emergence and global spread of resistant strains most notably oseltamivir-resistant H1N1 and H3N2 variants harboring the H274Y NA substitution poses a serious clinical challenge [5,6]. These limitations underscore the urgent need for novel antiviral agents with alternative mechanisms of action.

Natural products remain an important source of chemically diverse scaffolds for antiviral research, and complex botanical extracts may influence multiple viral or host-associated processes [7,8]. In traditional East Asian medicine, two distinct botanical species are associated with the name ‘Sokdan’: *Dipsacus asperoides* C.Y.Cheng & T.M.Ai and *Phlomis umbrosa* Turcz. According to the current Plants of the World Online taxonomic frame-work, *D. asperoides* is treated as a synonym of *Dipsacus asper* Wall. ex DC., and *P. umbrosa* as a synonym of *Phlomoides umbrosa* (Turcz.) Kamelin & Makhm [9,10]. For consistency with traditional, pharmacopoeial, and previous research usage, however, the names *D. asperoides* and *P. umbrosa* are retained throughout this manuscript. *D. asperoides* (family Caprifoliaceae), often referred to as ‘Cheon-Sokdan’, is the officially recognized source of Dipsaci Radix in the Korean and Chinese Pharmacopoeias, whereas *P. umbrosa* (family Lamiaceae), commonly known as ‘Han-Sokdan’, has historically been used for similar indications, including bone fractures, joint diseases, and inflammatory conditions [11,12]. Their distinct taxonomic origins and secondary-metabolite profiles make comparative botanical, chemical, and pharmacological characterization relevant to the appropriate interpretation of these medicinal materials.

Previous phytochemical studies of Dipsacus and Phlomis/Phlomoides-related medicinal materials have reported iridoid glycosides, phenylpropanoids, and triterpenoid saponins among their characteristic constituents [13,14]. These compound classes have been associated with anti-inflammatory, antioxidant, immunomodulatory, and, for selected constituents, antiviral effects [15]. However, the two Sokdan species have not been systematically compared in the same IAV model while their phytochemical differences are characterized in parallel. A comparative design therefore offers an opportunity to ask whether botanically and chemically distinct materials can nevertheless show a shared biological response under the same experimental conditions.

Network pharmacology integrates compound information, predicted targets, protein-interaction networks, and pathway enrichment to generate systems-level hypotheses for multicomponent herbal medicines [16,17]. Importantly, such analyses are predictive and are most appropriately used to prioritize candidate targets and pathways for experimental follow-up rather than to establish mechanism on their own. Recent herbal-medicine studies have similarly used integrated network-pharmacology workflows as hypothesis-generating frameworks [18].

Accordingly, the present study was designed to: (i) compare the morphology and qualitative LC-QTOF-MS chemical profiles of *D. asperoides* and *P. umbrosa*; (ii) compare their cytotoxicity, CPE-reduction responses against IAV A/PR/8/34 (H1N1), and NA inhibitory activity under the same in vitro framework; and (iii) explore whether the chemically distinct species-specific compound sets map to overlapping or distinct host-associated targets and pathways that may guide subsequent mechanistic validation. The network analysis was therefore treated as an exploratory, hypothesis-generating component of the study.

## 2. Results

### 2.1. Comparative Morphological Analysis and Taxonomic Differentiation of Dipsacus asperoides and Phlomis umbrosa

**Figure 1:**
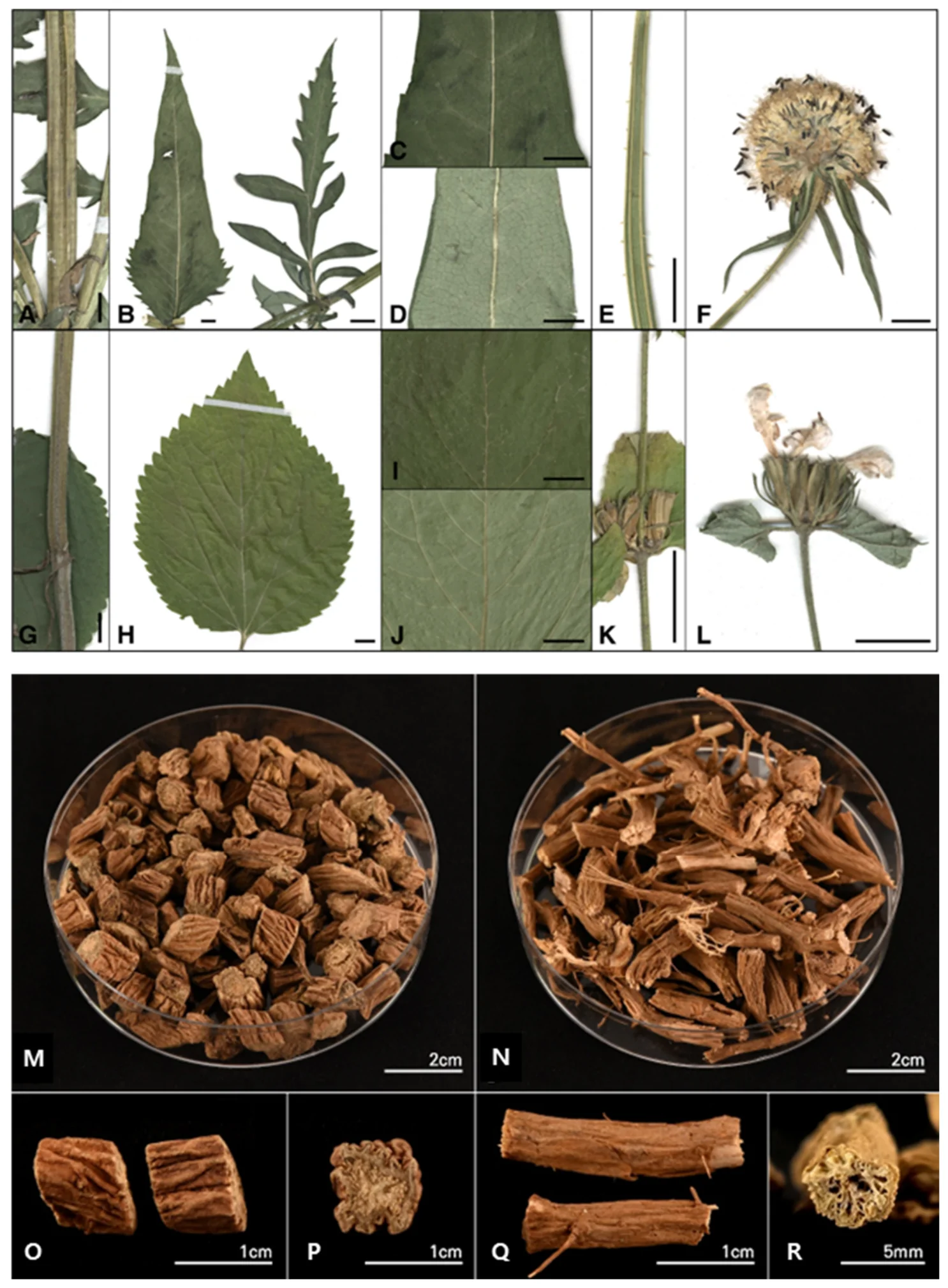
Comparative morphology of voucher specimens and macroscopic characteristics of the commercial crude drug materials used in this study. A–L, voucher specimens of *Dipsacus asperoides* (**A**–**F**) and *Phlomis umbrosa* (**G**–**L**): stem (**A**,**G**), leaf (**B**,**H**), adaxial leaf surface (**C**,**I**), abaxial leaf surface (**D**,**J**), peduncle (**E**,**K**), and inflorescence (**F**,**L**). (**M**–**R**), commercially sourced dried root specimens used for extract preparation: *D. asperoides* (**M**,**O**,**P**) and *P. umbrosa* (**N**,**Q**,**R**). General appearance (**M**,**N**), external root surface (**O**,**Q**), and transverse section (**P**,**R**). The crude drug materials shown in (**M**–**R**) were used to prepare the extracts subjected to LC-QTOF-MS chemical profiling and antiviral assays. Scale bars: (**A**–**L**), (**O**–**Q**), 1 cm; (**M**,**N**), 2 cm; (**R**), 5 mm.

The aerial morphology of *D. asperoides* and *P. umbrosa* clearly reflects their distinct taxonomic origins (Figure 1A–L). The stem of *D. asperoides* (Figure 1A) is angular and ridged, armed with scattered downward-curving prickles along the ridges, a diagnostic feature of the genus Dipsacus. In contrast, the stem of *P. umbrosa* (Figure 1G) is quadrangular in cross-section and densely pubescent, lacking prickles—a typical characteristic of the Lamiaceae.

The leaves also differ markedly. *D. asperoides* possesses deeply pinnatifid to lobed leaves with lanceolate, serrate segments (Figure 1B), whereas *P. umbrosa* has simple, broadly ovate cordate leaves with a coarsely serrate margin and a rugose (bullate) surface texture (Figure 1H). On the adaxial surface, *D. asperoides* shows a smooth, sparsely pubescent lamina with a slightly raised midrib (Figure 1C), while *P. umbrosa* exhibits a more strongly rugose, hairy adaxial surface with prominent reticulate venation (Figure 1I). The abaxial surfaces follow the same pattern: *D. asperoides* is pale green and glabrous to sparsely hairy along the veins (Figure 1D), whereas *P. umbrosa* is densely pubescent with conspicuous raised veins (Figure 1J), consistent with the tomentose character common in Lamiaceae foliage.

The peduncle of *D. asperoides* is long, erect, and armed with scattered prickles similar to the stem (Figure 1E), while that of *P. umbrosa* is shorter, pubescent, and subtends a whorled (verticillate) arrangement of flowers rather than a single terminal axis (Figure 1K). Finally, the inflorescence architecture provides the most distinctive contrast: *D. asperoides* forms a dense, globose head subtended by spiny, elongated involucral bracts (Figure 1F), typical of the former Dipsacaceae, whereas *P. umbrosa* produces a whorled verticillaster inflorescence with tubular calyces arranged in discrete axillary clusters (Figure 1L), a hallmark of the Lamiaceae.

Overall, these morphological differences in stem shape, leaf architecture, surface pubescence, and inflorescence type clearly distinguish *P. umbrosa* from *D. asperoides*, consistent with their placement in different families (Lamiaceae vs. Caprifoliaceae, formerly Dipsacaceae).

For the phytochemical and biological experiments, commercially sourced dried roots of the two Sokdan species were used as the crude drug materials (Figure 1M–R). Because *D. asperoides* is not naturally distributed in Korea, commercially available crude drug material was used as the experimental source of this species. The roots of *D. asperoides* exhibited distinct longitudinal wrinkles, some of which formed deep furrows, and their transverse sections showed a clear distinction between the cortex and stele (Figure 1M,O,P). In contrast, *P. umbrosa* roots showed relatively shallow longitudinal wrinkles with traces of fine roots on the surface, while their transverse sections exhibited prominent radially arranged fissures and intervening gaps (Figure 1N,Q,R). These macroscopic features were consistent with the descriptions provided in *Korean Medicinal Materials*, Vol. 1 [19], providing additional morphological support for the identity of the crude drug materials used for extract preparation and subsequent phytochemical and biological analyses.

### 2.2. Qualitative LC-QTOF-MS Chemical Profiling of Dipsacus asperoides and Phlomis umbrosa

**Figure 2:**
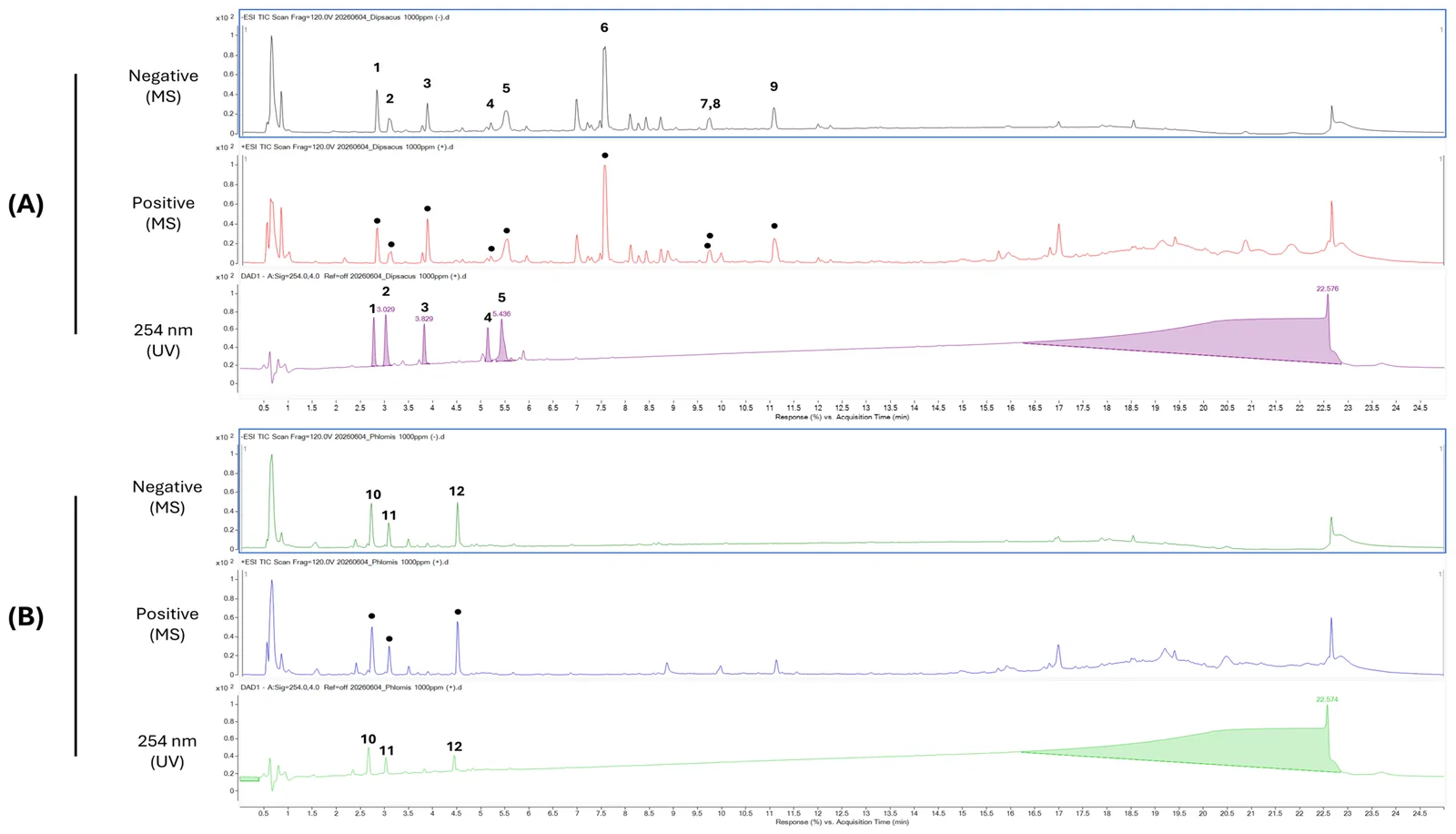
LC-QTOF-MS-based chemical profiling of *Dipsacus asperoides* (**A**) and *Phlomis umbrosa* (**B**). Upper traces: negative ESI-TIC chromatograms; middle traces: positive ESI-TIC chromatograms; lower traces: UV chromatograms at 254 nm. Identified compounds: loganic acid (**1**), chlorogenic acid (**2**), sweroside (**3**), isochlorogenic acid A (**4**), isochlorogenic acid C (**5**), akebia saponin D (**6**), dipsacus saponin C (**7**), dipsacus saponin B (**8**), akebia saponin PA (**9**), sesamoside (**10**), morroniside (**11**), and umbroside (**12**). Filled circles (●) denote MS-confirmed peaks.

Representative LC-QTOF-MS chromatograms of the ethanolic extracts of *D. asperoides* and *P. umbrosa* are shown in Figure 2A,B, respectively. A total of twelve compounds were annotated based on accurate mass, molecular formula, MS fragmentation patterns, retention behavior, and comparison with authentic reference standards and/or an in-house compound library. These included three phenylpropanoids (chlorogenic acid, isochlorogenic acids A and C), five iridoid glycosides (loganic acid, sweroside, sesamoside, morroniside, umbroside), and four triterpenoid saponins (akebia saponin D, akebia saponin PA, dipsacus saponins B and C). The identified compounds and their detection patterns are summarized in Table 1.

The chromatographic profiles showed both shared and species-associated constituents. Chlorogenic acid (**2**), isochlorogenic acids A (**4**) and C (**5**), akebia saponin D (**6**), akebia saponin PA (**9**), and sesamoside (**10**) were detected in both extracts, although several were recorded only at trace signal intensity in one of the species. Loganic acid (**1**), sweroside (**3**), dipsacus saponin C (**7**), and dipsacus saponin B (**8**) were detected only in *D. asperoides* under the present analytical conditions, whereas morroniside (**11**) and umbroside (**12**) were detected only in *P. umbrosa*. Thus, the two extracts were distinguishable by their qualitative constituent patterns. Because this LC-QTOF-MS experiment was designed for compositional profiling rather than concentration determination, chromatographic signal intensity was not used to infer quantitative abundance or a concentration-activity relationship.

### 2.3. Cytotoxicity of Dipsacus asperoides and Phlomis umbrosa Extracts in MDCK Cells

**Figure 3:**
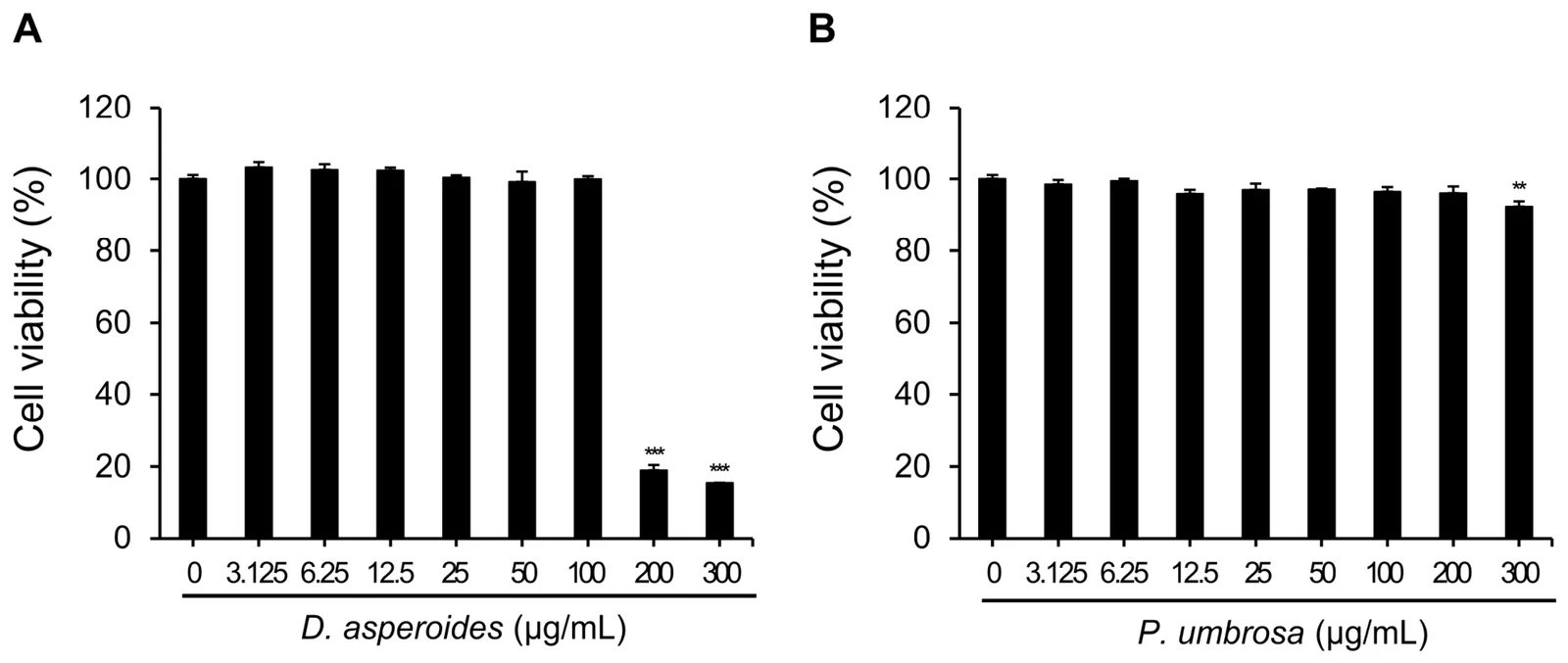
Cell viability of MDCK cells treated with *Dipsacus asperoides* (**A**) and *Phlomis umbrosa* (**B**) extracts. Cells were treated with various concentrations (3.125–300 μg/mL) for 48 h. Data are presented as mean ± SEM (*n* = 3). Statistical significance was analyzed using one-way ANOVA followed by Dunnett’s multiple-comparisons test. ** *p* < 0.01; *** *p* < 0.001 vs. untreated control.

The cytotoxicity of the extracts was evaluated in MDCK cells at concentrations ranging from 3.125 to 300 µg/mL (Figure 3). The *D. asperoides* extract exhibited no cytotoxic effects at concentrations up to 100 µg/mL, maintaining cell viability above 99.9%. However, it significantly reduced cell viability at 200 and 300 µg/mL (*p* < 0.001). In contrast, the *P. umbrosa* extract maintained high cell viability (>92.3%) across all tested concentrations, although a slight but statistically significant reduction was observed at 300 µg/mL (*p* < 0.01). Subsequent antiviral assays were performed at non-cytotoxic concentrations of *D. asperoides* extract (≤100 µg/mL) and *P. umbrosa* extract (≤300 µg/mL).

### 2.4. Modest Antiviral Responses Against Influenza A Virus (Cytopathic Effect Reduction Assay)

**Figure 4:**
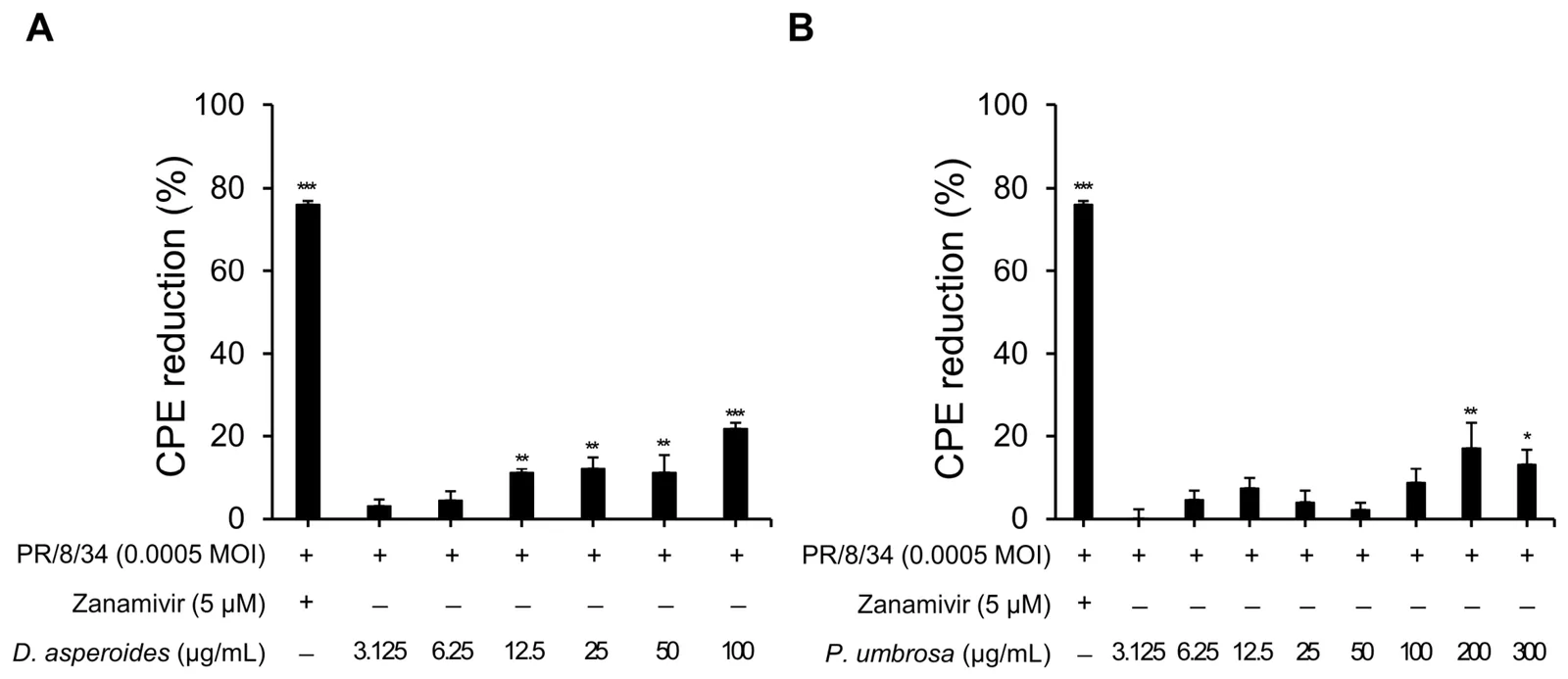
CPE-reduction responses of *Dipsacus asperoides* (**A**) and *Phlomis umbrosa* (**B**) extracts against influenza A virus A/PR/8/34 (H1N1). MDCK cells were infected at an MOI of 0.0005 and treated with the indicated extract concentrations. Zanamivir (5 µM) was included as a positive control and produced 76.2 ± 0.8% inhibition under the same assay conditions. Data are presented as mean ± SEM (*n* = 3). Statistical significance was analyzed using one-way ANOVA followed by Dunnett’s multiple-comparisons test. Statistical symbols indicate comparisons with the virus-infected untreated control: * *p* < 0.05; ** *p* < 0.01; *** *p* < 0.001. (–) denotes the single concentration tested for the positive control.

The antiviral activities of both extracts against IAV A/PR/8/34 (H1N1) are summarized in Figure 4. Infection of MDCK cells at an MOI of 0.0005 produced pronounced CPE in the untreated virus control. Under the same assay conditions, the positive control zanamivir (5 µM) produced 76.2 ± 0.8% virus inhibition, confirming that the assay detected a robust antiviral response to a standard drug.

*D. asperoides* produced a concentration-related increase in CPE reduction over the non-cytotoxic concentration range, from 3.1 ± 1.5% at 3.125 µg/mL to a maximum of 21.8 ± 1.4% at 100 µg/mL. Significant differences from the virus control were observed at concentrations ≥12.5 µg/mL (*p* < 0.01). However, the maximum inhibition remained substantially below that of zanamivir and should therefore be interpreted as a modest in vitro response rather than high antiviral potency. *P. umbrosa* also showed partial CPE reduction, reaching 17.1 ± 6.1% at 200 µg/mL and 13.2 ± 3.6% at 300 µg/mL. Significant differences from the virus control were observed at 200 µg/mL (*p* < 0.01) and 300 µg/mL (*p* < 0.05). Because inhibition did not increase further at 300 μg/mL, a consistent concentration-dependent response was not observed for *P. umbrosa* across the entire tested range. Taken together, the two extracts showed a broadly similar pattern of modest inhibition in this assay, despite their different phytochemical profiles. Because the statistical tests reported here compare each treatment with the virus control rather than directly testing the two species against each other, the present data are not used to claim a statistically established difference in antiviral potency between the species.

### 2.5. Neuraminidase Inhibitory Activity

**Figure 5:**
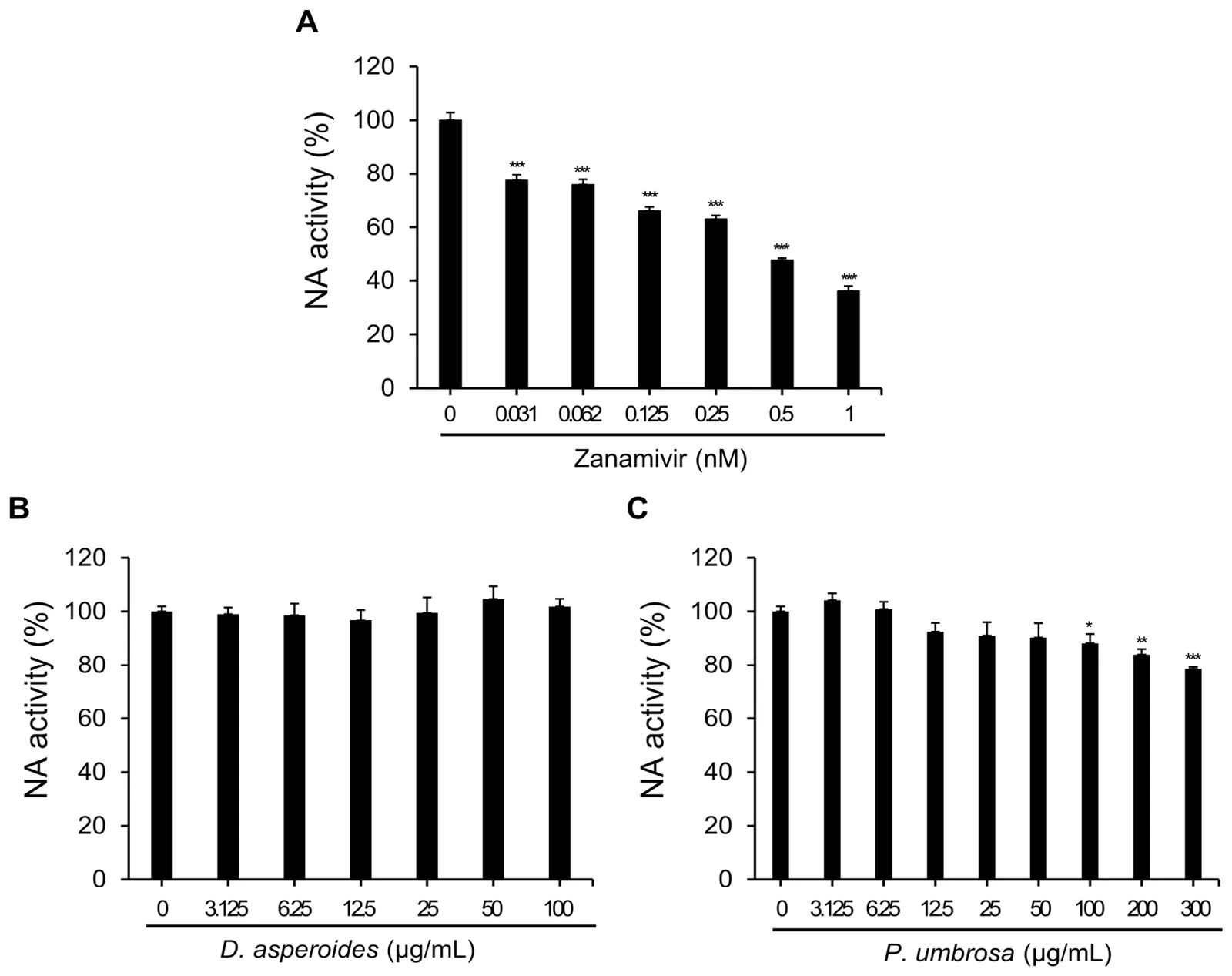
Neuraminidase inhibitory activity of zanamivir (**A**), *Dipsacus asperoides* (**B**), and *Phlomis umbrosa* (**C**). Influenza A virus NA activity was measured using the NA-Star™ chemiluminescent assay. A/PR/8/34 virus was exposed to *D. asperoides* extract (3.125–100 μg/mL) or *P. umbrosa* extract (3.125–300 μg/mL) for 30 min before determination of NA activity. Zanamivir (0.031–1 nM) was included as a positive control. Data are presented as mean ± SEM. Statistical significance was analyzed using one-way ANOVA followed by Dunnett’s multiple-comparisons test. * *p* < 0.05; ** *p* < 0.01; *** *p* < 0.001 vs. untreated virus control.

The NA inhibitory activities of the extracts were examined to determine whether direct inhibition of viral NA could account for the CPE-reduction response (Figure 5). Both *D. asperoides* and *P. umbrosa* showed limited NA inhibition across the tested concentration ranges, with inhibition remaining below 21.5%, whereas zanamivir showed potent NA inhibition (IC_50_ ≈ 0.4 nM). These findings indicate that direct NA inhibition is unlikely to be the principal explanation for the modest CPE-reduction responses of either extract. They do not, however, establish a specific alternative mechanism, which requires targeted experimental validation.

### 2.6. Exploratory Network Pharmacology: Predicted Targets and Convergent Pathways

#### 2.6.1. Predicted Target Profile of *Dipsacus asperoides*

The *D. asperoides* compound set used for exploratory target prediction comprised loganic acid, chlorogenic acid, sweroside, isochlorogenic acids A and C, akebia saponin D, akebia saponin PA, and dipsacus saponins B and C. Predicted human targets from SwissTargetPrediction and PharmMapper were intersected with IAV-related disease targets from DisGeNET and GeneCards, and the resulting candidates were analyzed using STRING and Cytoscape. The *D. asperoides* network included AKT1, TP53, TNF, IL-6, MAPK1/MAPK3, RELA, TLR4, and CASP3 among the topologically prominent predicted hubs (Table 2).

These outputs were interpreted as candidate network associations rather than evidence of direct target engagement. For continuity with the original analysis, the literature reports were used only to contextualize the predicted links summarized in Table 2. Chlorogenic acid and isochlorogenic acids have been associated with PI3K/Akt- or TLR4/NF-κB-related responses, including antiviral or inflammation-related models [20,21,22]; sweroside and chlorogenic acid have been reported as candidate anti-influenza constituents in a network-pharmacology study of Shuang-Huang-Lian [23]; akebia saponin D has been linked to PI3K/Akt and IL-6/STAT3 signaling [24,25]; and akebia saponin PA has been associated with MAPK- and caspase-related apoptosis [26]. Dipsacus saponin C has mainly been characterized in antinociceptive models [27,28], and its relevance to IAV remains unresolved. Several predicted hubs in the *D. asperoides* network—MAPK1/3, RELA, IL-6, TNF, and CASP3—were also present in the *P. umbrosa* network, whereas AKT1, TP53, and TLR4 were specific to the *D. asperoides* prediction under the applied input and filtering criteria. These literature citations provide biological context only and are not treated as experimental validation of target engagement in the present MDCK-IAV model.

#### 2.6.2. Predicted Target Profile of *Phlomis umbrosa*

The *P. umbrosa* compound set used for target prediction comprised chlorogenic acid, sesamoside, morroniside, and umbroside; compounds detected only at trace signal intensity were excluded according to the predefined input criterion. Following the same target-prediction and disease-target intersection workflow, the *P. umbrosa* PPI network highlighted MAPK1/MAPK3, RELA, IL-6, TNF, and CASP3 as predicted hubs (Table 2).

Comparison of the two species-specific networks revealed partial convergence on MAPK, NF-κB/cytokine-associated, and apoptosis-related nodes despite the different compound inputs. Morroniside, a constituent specific to the *P. umbrosa* profile in the present analysis, has previously been associated with NF-κB/IL-6/TNF responses and JNK/p38 MAPK signaling in non-viral models [29,30]. The *D. asperoides* network additionally contained predicted AKT1, TP53, and TLR4 connections. These findings generate a testable hypothesis that chemically distinct constituent sets may converge on overlapping host-response pathways while retaining species-specific network features. Because no target or pathway was experimentally measured in the infected MDCK-cell model, the network results are not considered mechanistic confirmation.

**Table 2:** (**A**) Hub targets and predicted interacting compounds of *Dipsacus asperoides*. (**B**) Hub targets and predicted interacting compounds of *Phlomis umbrosa*.

(**A**)			
**Hub** **Target**	**Key Pathway**	**Predicted Interacting Compounds**	**Supporting Evidence**
AKT1	PI3K/Akt signaling	Chlorogenic acid, Isochlorogenic A/C, Akebia saponin D	[20,24,31]
TP53	Apoptosis, Cell cycle	Sweroside, Chlorogenic acid	[32,33]
TNF	TNF signaling, NF-κB	Chlorogenic acid, Isochlorogenic A/C,	[33,34]
IL-6	Cytokine signaling	Loganic acid, Isochlorogenic C, Akebia saponin D	[25,34,35]
MAPK1/MAPK3	MAPK, Influenza A pathway	Sweroside, Chlorogenic acid, Akebia saponin PA	[23,26,33]
RELA	NF-κB signaling	Isochlorogenic acid A/C	[34]
TLR4	Innate immunity, Influenza A	Isochlorogenic acid A	[22]
CASP3	Apoptosis	Sweroside, Isochlorogenic A, Akebia saponin PA	[26,31,32]
(**B**)			
**Hub** **Target**	**Key Pathway**	**Predicted Interacting Compounds**	**Supporting Evidence**
MAPK1/MAPK3	MAPK, Influenza A pathway	Morroniside, Umbroside	[30]
RELA	NF-κB signaling	Morroniside	[29]
IL-6	Cytokine signaling	Morroniside	[29]
TNF	TNF signaling, NF-κB	Morroniside	[29]
CASP3	Apoptosis	Sesamoside	—
AKT1, TP53, TLR4	—	No interacting compound retained under the applied prediction/input criteria	Species-associated prediction; not evidence of lower antiviral potency

Dipsacus saponin B, dipsacus saponin C: no documented hub-target connectivity pending direct prediction.

## 3. Discussion

The principal finding of this study is not the identification of a highly potent antiviral extract, but the observation that two botanically and chemically distinct medicinal materials traditionally used as ‘Sokdan’ show a shared pattern of modest in vitro response to IAV under the same experimental framework. To our knowledge, this is the first study to compare *D. asperoides* and *P. umbrosa* by integrating morphological differentiation, qualitative LC-QTOF-MS profiling, CPE-reduction and NA assays, and an exploratory network-pharmacology comparison. This integrated design allows the chemical divergence of the two species to be considered alongside a partially convergent biological phenotype.

The CPE assay places the magnitude of the observed antiviral response in a clear context. At the low viral inoculum used in this study (MOI 0.0005), *D. asperoides* and *P. umbrosa* reached maximum inhibition of 21.8% and 17.1%, respectively, whereas zanamivir produced 76.2% inhibition under the same conditions. Thus, neither crude extract should be interpreted as a high-potency anti-IAV agent on the basis of the present data. The value of the result lies instead in the reproducible direction of the response across two different Sokdan species and in the opportunity to use their contrasting chemical profiles to formulate questions for subsequent fractionation and mechanism-focused studies.

The LC-QTOF-MS data support clear phytochemical differentiation between the two species. The profiles contained both shared constituents and species-associated detection patterns, with loganic acid, sweroside, and dipsacus saponins B/C detected only in *D. asperoides* under the applied conditions and morroniside and umbroside detected only in *P. umbrosa*. Several phenylpropanoids and akebia saponins were shared but occurred at trace signal intensity in *P. umbrosa*, whereas sesamoside was prominent in the *P. umbrosa* profile and only trace in *D. asperoides*. Previous comparative studies have likewise emphasized the botanical and pharmacological distinction between the two Sokdan materials [12,14]. The purpose of this LC-QTOF-MS component was qualitative compositional characterization; therefore, the chromatograms are not used to infer absolute or relative concentrations, and no concentration-based structure-activity relationship is claimed. This distinction is important because the central comparison concerns chemical identity and profile pattern rather than quantitative marker standardization.

Several constituents identified in the two extracts have prior biological evidence relevant to antiviral or host-response pathways. Chlorogenic acid has reported anti-IAV activity and has been linked to effects on viral replication and host PI3K/Akt signaling [21,36]. Sweroside and chlorogenic acid have also appeared as candidate components in an anti-influenza network-pharmacology study of Shuang-Huang-Lian [23]. Isochlorogenic acid A has shown antiviral activity in a hepatitis B virus model [37], and isochlorogenic acid C has been implicated in the anti-respiratory syncytial virus activity of Lonicera japonica [38]. Morroniside has been associated with NF-κB, IL-6, TNF, and MAPK-related responses in non-viral inflammatory models [29,30]. These virus systems and experimental contexts differ from the present IAV-MDCK model and therefore provide biological context rather than direct validation. More broadly, plant polyphenols are increasingly discussed as multitarget antimicrobial scaffolds, although potency, bioavailability, and translational validation remain major constraints [39].

The lack of substantial NA inhibition in either extract further refines, but does not resolve, the mechanistic question. Plant-derived extracts can affect IAV through processes other than direct NA inhibition [40]. Because the CPE-reduction response in the present study was not accompanied by strong direct NA inhibition, the observed cellular response may involve other viral processes, host-response pathways, or a combination of effects. The current data do not distinguish among these possibilities, and the NA result should therefore be interpreted as excluding a dominant NA-inhibitory explanation rather than demonstrating a specific host-directed mechanism.

In this context, network pharmacology is most useful as a hypothesis-generating bridge between chemical profiling and future mechanistic experiments. The two species-specific networks partially converged on MAPK1/3, RELA, IL-6, TNF, and CASP3, while the *D. asperoides* prediction additionally included AKT1, TP53, and TLR4. Host-signaling candidates are biologically plausible in IAV because viral NS1 can activate PI3K/Akt signaling [36,41] and interfere with innate antiviral sensing such as RIG-I-mediated responses [42]. However, this biological plausibility does not demonstrate that the predicted nodes were modulated by either extract. Integrated network-pharmacology workflows have been used to prioritize candidate mechanisms in herbal-product research [18], and a recent study of Centipeda minima provides an additional example of network pharmacology applied to a traditional herbal medicine [43]. Accordingly, network topology and enrichment in the present study are interpreted only as a basis for prioritizing subsequent experimental validation.

Accordingly, the network results define specific directions for follow-up rather than a completed mechanism. A focused next step would be to test one or two shared predicted axes in the same IAV-MDCK model—for example, MAPK/ERK and NF-κB-related responses—together with viral replication markers, and then determine whether activity tracks with specific fractions or purified constituents. Species-associated predictions such as AKT1 or TLR4 could subsequently be examined if supported by fractionation or target-level data. This staged approach would directly test whether the chemical divergence observed by LC-QTOF-MS converges functionally at the pathway level.

The study should also be interpreted within several experimental boundaries. First, the CPE-reduction effect of both crude extracts was modest relative to zanamivir, even at the low MOI used, so therapeutic or translational claims are premature. Second, the network-pharmacology component is predictive and requires experimental validation before any target or pathway can be considered mechanistically involved. Third, the current LC-QTOF-MS analysis was intentionally designed as qualitative profiling and does not provide concentration-based comparisons; accordingly, the present manuscript does not infer antiviral potency from compound abundance. Future work combining bioactivity-guided fractionation, targeted quantitative analysis of prioritized fractions or constituents, and pathway-level validation would be more appropriate once active chemical entities are narrowed.

Taken together, the results support a revised interpretation of the two Sokdan species: they are clearly distinguishable botanically and by qualitative phytochemical profile, yet both produced a comparable pattern of modest CPE reduction against IAV in the present model. The exploratory network analysis suggests that distinct compound sets may map partly to shared host-response nodes, offering a mechanistic hypothesis for future testing rather than a demonstrated explanation of activity. The scientific value of the study therefore lies in integrating chemical differentiation with a shared biological response and in defining experimentally testable directions for subsequent antiviral research.

## 4. Materials and Methods

### 4.1. Plant Materials, Taxonomic Identification, and Extract Preparation

Voucher specimens of *Dipsacus asperoides* C.Y. Cheng & T.M. Ai and *Phlomoides umbrosa* (Turcz.) Kamelin & Makhm were used for comparative morphological characterization. Two voucher specimens of *D. asperoides* (voucher Nos. KIOM201701018837 and KIOM201201004804) were collected from the Agricultural Research and Extension Services, Cheongju-si, Chungcheongbuk-do, Republic of Korea, on 18 July 2016 by S. Yang, and from the Agricultural Seedling Station, Jeju-si, Jeju-do, Republic of Korea, on 7 August 2012 by Y. Ji and B. C. Moon, respectively. One voucher specimen of *P. umbrosa* (voucher No. KIOM201301005989) was collected from Seorak-myeon, Gapyeong-gun, Gyeonggi-do, Republic of Korea, on 25 June 2013 by B. C. Moon and Y. Ji. All voucher specimens are deposited in the Herbarium of the Korea Institute of Oriental Medicine (KIOM), Naju, Republic of Korea.

The crude drug materials used for phytochemical and biological analyses consisted of commercially sourced dried roots of *D. asperoides* (voucher No. 2-17-0065, KIOM) and *P. umbrosa* (voucher No. 2-17-0072, KIOM). Their identities were confirmed by comparison with the corresponding voucher specimens and with the diagnostic descriptions provided in the Korean Pharmacopoeia and *Korean Medicinal Materials*, Vol. 1. Representative authenticated specimens of these commercial crude drug materials are deposited in the Herbal Medicine Resources Research Center, Korea Institute of Oriental Medicine (KIOM), Naju, Republic of Korea. The authenticated dried root materials shown in Figure 1M–R were used to prepare the extracts subjected to LC-QTOF-MS chemical profiling and all biological assays in this study.

Dried root material (100 g of each species) was extracted twice under reflux with 1 L of 70% aqueous ethanol (plant material-to-solvent ratio, 1:10, *w*/*v*) for 2 h per extraction. The combined extracts were filtered, concentrated under reduced pressure using a rotary evaporator, and lyophilized to obtain dry extracts, yielding 47.86% (*w*/*w*) for *D. asperoides* and 26.62% (*w*/*w*) for *P. umbrosa*. For LC-QTOF-MS analysis, the dried extracts were dissolved in 70% methanol at 1 mg/mL and filtered through a 0.22 μm PTFE syringe filter prior to analysis. For biological assays, dried extracts were dissolved in dimethyl sulfoxide (DMSO; Sigma-Aldrich, St. Louis, MO, USA) to prepare stock solutions (100 mg/mL) and diluted with culture medium to the desired working concentrations. The final DMSO concentration in all assays was ≤0.1% (*v*/*v*).

### 4.2. Cell Culture and Virus

Madin–Darby canine kidney (MDCK) cells (ATCC CCL-34) were maintained in Eagle’s Minimum Essential Medium (EMEM; Welgene, Gyeongsan, Korea) supplemented with 10% heat-inactivated fetal bovine serum (FBS; Gibco, Grand Island, NY, USA), 100 U/mL penicillin, and 100 μg/mL streptomycin (Gibco) at 37 °C in a humidified 5% CO_2_ atmosphere. Influenza A virus (A/Puerto Rico/8/1934; H1N1; hereafter A/PR/8/34) was propagated in MDCK cells, titrated by plaque assay, and stored as aliquots at −80 °C until use.

### 4.3. Cytotoxicity Assay

MDCK cells were seeded in 96-well plates (2.5 × 10^4^ cells/well) and allowed to adhere overnight. Cells were then treated with serial dilutions of each extract (3.125–300 μg/mL) for 48 h. Cell viability was determined by the MTT [3-(4,5-dimethylthiazol-2-yl)-2,5-diphenyltetrazolium bromide] reduction assay. Briefly, MTT solution (5 mg/mL in PBS; Sigma-Aldrich, St. Louis, MO, USA) was added to each well and incubated for 4 h at 37 °C. The resulting formazan crystals were dissolved in DMSO, and absorbance was measured at 570 nm using a microplate reader (SpectraMax iD3, Molecular Devices, San Jose, CA, USA). Cell viability was expressed as a percentage relative to untreated controls.

### 4.4. Antiviral Activity Assay (CPE Reduction)

Antiviral activity was assessed by a CPE reduction assay. MDCK cells were seeded in 96-well plates and, after overnight adhesion, infected with A/PR/8/34 at a multiplicity of infection (MOI) of 0.0005 for 1 h at 37 °C. The viral inoculum was removed, and the cells were immediately treated with *D. asperoides* extract (3.125–100 μg/mL) or *P. umbrosa* extract (3.125–300 μg/mL) in serum-free EMEM supplemented with 2 μg/mL tosylsulfonyl phenylalanyl chloromethyl ketone (TPCK)-trypsin (Sigma-Aldrich, St. Louis, MO, USA). After 48 h of incubation, cell viability was determined by MTT assay as described above. Zanamivir (Relenza; GlaxoSmithKline, Brentford, UK) at 5 μM was used as a positive control. Antiviral activity was expressed as the percentage virus inhibition calculated relative to virus-infected, untreated controls.

### 4.5. Neuraminidase Inhibition Assay

NA inhibitory activity was determined using a fluorescence-based NA-Star Influenza Neuraminidase Inhibitor Resistance Detection Kit (Applied Biosystems, Waltham, MA, USA) according to the manufacturer’s instructions. Briefly, the A/PR/8/34 virus (MOI of 0.0005) was incubated with *D. asperoides* extract (3.125–100 μg/mL) or *P. umbrosa* extract (3.125–300 μg/mL) for 30 min at room temperature. The NA-Star substrate was added, and reactions were incubated for 30 min at 37 °C. Luminescence was measured using a microplate luminometer (GloMax Navigator, Promega, Madison, WI, USA). Zanamivir (0.031–1 nM) served as a positive control.

### 4.6. LC-QTOF-MS Chemical Profiling

LC–QTOF–MS analysis was performed using an Agilent 1290 Infinity II UHPLC system coupled with an Agilent 6546 Q-TOF mass spectrometer equipped with a Jet Stream electrospray ionization (ESI) source (Agilent Technologies, Santa Clara, CA, USA). Chromatographic separation was achieved using an ACQUITY UPLC BEH C18 column (2.1 × 100 mm, 1.7 μm; Waters, Milford, MA, USA). A 1 μL aliquot of each sample was injected, and separation was performed at a flow rate of 0.4 mL/min using a binary mobile phase consisting of 0.1% formic acid in water (A) and 0.1% formic acid in acetonitrile (B). The gradient program was as follows: 5% B (0–1 min), 5–100% B (1–20.0 min), 100% B (20.0–22.3 min), 100–5% B (22.3–22.4 min), and 5% B (22.4–25.0 min). The column and autosampler temperatures were maintained at 35 °C and 15 °C, respectively.

Mass spectrometric data were acquired in both positive and negative ion modes over an *m*/*z* range of 100–1700 using the MS acquisition mode. The optimized source parameters were as follows: drying gas temperature, 250 °C; drying gas flow, 10 L/min; nebulizer pressure, 35 psi; sheath gas temperature, 350 °C; sheath gas flow, 11 L/min; capillary voltage, ±4500 V; nozzle voltage, ±1000 V; fragmentor voltage, 120 V; and skimmer voltage, 65 V. Mass spectra were acquired at a rate of 1 spectrum/s. Continuous mass calibration was performed using Agilent reference mass solution. Data acquisition and processing were carried out using MassHunter software (version 10.0; Agilent Technologies).

Compounds were identified based on accurate mass measurements, molecular formula prediction, retention time, and MS fragmentation patterns through comparison with authentic reference standards and an in-house compound library. The occurrence of each identified compound in *D. asperoides* and *P. umbrosa* extracts was subsequently evaluated based on the corresponding LC-QTOF-MS chromatograms.

### 4.7. Exploratory Network Pharmacology Analysis

#### 4.7.1. Target Prediction for Identified Compounds

The twelve compounds annotated by LC-QTOF-MS (Table 1) were divided into species-specific input sets according to their qualitative detection patterns. The *D. asperoides* input set included loganic acid, chlorogenic acid, sweroside, isochlorogenic acids A and C, akebia saponin D, akebia saponin PA, and dipsacus saponins B and C. The *P. umbrosa* input set included chlorogenic acid, sesamoside, morroniside, and umbroside; compounds detected only at trace signal intensity in a given species were excluded from that species’ primary input set. Each set was analyzed separately using SwissTargetPrediction and PharmMapper to predict human protein targets. Disease-associated targets for ‘influenza A infection’ and ‘viral infectious disease’ were retrieved from DisGeNET and GeneCards, and intersecting targets were retained as candidates. This workflow was used as a hypothesis-generating strategy consistent with established network-pharmacology approaches for multicomponent herbal products [16,17,18].

#### 4.7.2. PPI Network Construction and Hub Target Identification

Candidate targets for each species were imported separately into the STRING database (https://string-db.org; version 12.0; Homo sapiens; minimum interaction score ≥ 0.7) to construct two species-specific protein–protein interaction (PPI) network. The resulting network was visualized and analyzed in Cytoscape (version 3.10.1). Hub targets were identified by topological analysis using the NetworkAnalyzer plug-in, selecting nodes with degree, betweenness centrality, and closeness centrality values above the network medians, independently for the *D. asperoides* and *P. umbrosa* networks.

#### 4.7.3. GO and KEGG Pathway Enrichment Analysis

Gene Ontology (GO) enrichment and Kyoto Encyclopedia of Genes and Genomes (KEGG) pathway enrichment analyses were performed separately for each species using the DAVID bioinformatics platform. Pathways with Benjamini–Hochberg-corrected *p* values <0.05 were considered enriched. Compound-target-pathway networks were visualized in Cytoscape to compare predicted shared and species-associated pathway patterns. All network outputs were interpreted as computational predictions requiring experimental validation.

### 4.8. Statistical Analysis

Statistical analyses were performed using SigmaPlot 10.0 software (Systat Software Inc., San Jose, CA, USA). In vitro experiments, including cytotoxicity, antiviral, and neuraminidase inhibition assays, were performed in three independent experiments (*n* = 3), and the data are presented as the mean ± SEM. Before parametric analysis, the individual observations, residual distributions, and homogeneity of variance were examined for potential violations of the assumptions underlying one-way ANOVA. No clear violations were identified; therefore, statistical significance was evaluated using one-way ANOVA followed by Dunnett’s multiple-comparisons test, with each treatment group compared with the corresponding control group. Statistical significance was defined as an adjusted *p* value < 0.05.

## 5. Conclusions

*Dipsacus asperoides* and *Phlomis umbrosa* were clearly distinguishable by morphology and qualitative LC-QTOF-MS chemical profiles, yet both produced a shared pattern of modest CPE reduction against IAV A/PR/8/34 (H1N1) under the tested conditions. Maximum inhibition was 21.8% for *D. asperoides* and 17.1% for *P. umbrosa*, substantially below the 76.2% inhibition produced by zanamivir, and neither extract showed strong direct NA inhibition. Exploratory network pharmacology identified partially overlapping predicted host-response nodes, including MAPK1/3, RELA, IL-6, TNF, and CASP3, together with species-associated predictions. These computational results do not establish antiviral mechanism but provide testable hypotheses for pathway-level validation and bioactivity-guided chemical follow-up. The study therefore supports phytochemical differentiation of the two traditional Sokdan species while showing that distinct chemical profiles can be accompanied by a similar modest antiviral response in vitro.

## Figures and Tables

**Table 1:** Qualitative LC-QTOF-MS characterization of constituents detected in *Dipsacus asperoides* and *Phlomis umbrosa*. (+, detected; T, detected at trace signal intensity; −, not detected). The presence/trace notation is used for qualitative profiling and does not represent quantitative concentration differences.

No.	Compound Name	RT (min)	Negative *m/z*, Adduct	Positive *m/z*, Adduct	MW	Molecular Formula	Species	
							*D. asperoides*	*P. umbrosa*
1	Loganic acid	2.86	375.1289, [M-H]^−^	394.1707, [M+NH_4_]^+^	376.14	C_16_H_24_O_10_	+	−
2	Chlorogenic acid	3.09	353.0875, [M-H]^−^	355.1022, [M+H]^+^	354.1	C_16_H_18_O_9_	+	+ *
3	Sweroside	3.89	403.1244, [M+HCOO]^−^	359.1337, [M+H]^+^	358.13	C_16_H_22_O9	+	−
4	Isochlorogenic acid A	5.21	515.1187, [M-H]^−^	517.1337, [M+H]^+^	516.13	C_25_H_24_O_12_	+	T **
5	Isochlorogenic acid C	5.49	515.1187, [M-H]^−^	517.1337, [M+H]^+^	516.13	C_25_H_24_O_12_	+	T **
6	Akebia saponin D	7.58	973.5021, [M+HCOO]^−^	951.4924, [M+Na]^+^	928.5	C_47_H_76_O_18_	+	T **
7	Dipsacus saponin C	9.71	1351.6485, [M-H]^−^	1353.6685, [M+H]^+^	1352.66	C_64_H_104_O_30_	+	−
8	Dipsacus saponin B	9.76	1219.6256, [M-H]^−^	1221.6253, [M+H]^+^	1220.62	C_59_H_96_O_26_	+	−
9	Akebia saponin PA	11.08	649.395, [M+HCOO]^−^	627.3868, [M+Na]^+^	604.4	C_35_H_56_O_8_	+	T **
10	Sesamoside	2.73	465.1248, [M+HCOO]^−^	443.1160, [M+Na]^+^	420.13	C_17_H_24_O_12_	T **	+
11	Morroniside	3.08	451.1453, [M+HCOO]^−^	429.1375, [M+Na]^+^	406.15	C_17_H_26_O_11_	−	+
12	Umbroside	4.52	493.1562, [M+HCOO]^−^	471.1474, [M+Na]^+^	448.16	C_19_H_28_O_12_	−	+

* Overlap with other peak; ** T: trace indicates compounds detected with low signal intensity.

## Data Availability

The raw data supporting the conclusions of this article will be made available by the authors on request.
